# How Well Does the World Health Organization Definition of Domestic Violence Work for India?

**DOI:** 10.1371/journal.pone.0120909

**Published:** 2015-03-26

**Authors:** Ameeta S. Kalokhe, Ratnaprabha R. Potdar, Rob Stephenson, Kristin L. Dunkle, Anuradha Paranjape, Carlos del Rio, Seema Sahay

**Affiliations:** 1 Emory University School of Medicine, Department of Medicine, Division of Infectious Diseases, Atlanta, Georgia, United States of America; 2 Emory University Rollins School of Public Health, Department of Global Health, Atlanta, Georgia, United States of America; 3 National AIDS Research Institute, Pune, Maharashtra, India; 4 Emory University Rollins School of Public Health, Department of Behavioral Sciences and Health Education, Atlanta, Georgia, United States of America; 5 Temple University School of Medicine, Department of Medicine, Section of General Internal Medicine, Philadelphia, Pennsylvania, United States of America; Medical University of Vienna, AUSTRIA

## Abstract

Domestic violence (DV) is reported by 40% of married women in India and associated with substantial morbidity. An operational research definition is therefore needed to enhance understanding of DV epidemiology in India and inform DV interventions and measures. To arrive at a culturally-tailored definition, we aimed to better understand how definitions provided by the World Health Organization and the 2005 India Protection of Women from Domestic Violence Act match the perceptions of behaviors constituting DV among the Indian community. Between September 2012 and January 2013, 16 key informant interviews with experts in DV and family counseling and 2 gender-concordant focus groups of lay community members were conducted in Pune, India to understand community perceptions of the definition of DV, perpetrators of DV, and examples of DV encountered by married women in Pune, India. Several key themes emerged regarding behaviors and acts constituting DV including 1) the exertion of control over a woman’s reproductive decision-making, mobility, socializing with family and friends, finances, and access to food and nutrition, 2) the widespread acceptance of sexual abuse and the influences of affluence on sexual DV manifestations, 3) the shaping of physical abuse experiences by readily-available tools and the presence of witnesses, 4) psychological abuse for infertility, dowry, and girl-children, and 5) the perpetration of DV by the husband and other members of his family. Findings support the need for a culturally-tailored operational definition that expands on the WHO surveillance definition to inform the development of more effective DV intervention strategies and measures.

## Introduction

Approximately 40% of Indian women report lifetime physical, sexual, or psychological domestic violence according to recent national estimates [1]. Such acts of domestic violence (DV) have been linked to substantial mental and physical morbidity [2–20]. Indian women who report DV have a higher likelihood of having depression, post traumatic stress disorder, attempted suicide, and adopting maladaptive health behaviors [2–6,15]. They also report higher frequencies of injuries[16], chronic diseases such as asthma and anemia, gynecologic morbidity[18,19], and infectious diseases such as sexually transmitted infections and HIV [7–10,20]. Furthermore, they are more likely to have terminated unintended pregnancies and utilize less prenatal care and breastfeeding; and, their children have a greater probability of being malnourished, developing asthma, being insufficiently vaccinated, and dying early [11–15,17,21].

In recent years, recognition of the high prevalence of DV and the associated morbidity and mortality has fueled community and government momentum in India to develop enhanced strategies to curb its occurrence and increase support for women experiencing DV. In 2005 the Government of India passed the 2005 Protection of Women from Domestic Violence Act (PWDVA)[22]. The PWDVA was drafted using the input of multi-disciplinary community experts who provide DV support services and provided a framework for DV legal proceedings. It called for the appointment of female protection officers to serve as links between the women, the court systems, and community support services, led to the conception of DV protection orders, residence orders, and custody orders, and provided assurance that law enforcement and judicial service providers would receive periodic training in the use of the act. More recently, the Ministry of Women and Child Development released the 5-year Strategic Plan which proposed the development of ‘one-stop crisis centers’ that would provide medical, legal, counseling, and shelter support for female DV survivors and their children [23]. The Indian media has also been committed to raising DV awareness through daily publication of DV cases in the press and dedication of episodes in TV serial to gender-based violence [24,25]. Lastly, at the community-level, DV hotlines, women’s empowerment NGOs, shelters, hostels for working women, social advocacy campaigns, and women-led initiatives are expanding.

To continue the forward progress, consensus on an operational definition of DV that embraces community perceptions of DV and captures the full extent of DV in India is critical. Such a definition is essential for more refined research that enhances understanding of DV epidemiology in India, and thus would lead to culturally-tailored prevention strategies and more effective measures that evaluate the effectiveness of such interventions. In the 2005 Multi-country DV study, the World Health Organization (WHO) operational definition included acts of physical, sexual, psychological abuse, and control by an intimate partner during a woman’s lifetime [26]. In subsequent publications, the WHO continued to limit its survey of DV to that perpetrated by intimate partners, but acknowledged that DV could include behaviors perpetrated by other members of a household in some countries [27,28]. In India, no country-specific operational definition of DV has been developed or used, but the PWDVA provides a legal construct that incorporates several cultural considerations. In addition to the aforementioned forms of abuse, it devotes particular focus to economic abuse (i.e. dowry and *stridhan* (material assets given at the time of marriage by her parents)), broadens the definition to abuse perpetrated by *any* member of a shared household against the woman, and defines violence in terms of the physical, mental, psychological, or economic harm resulting to the woman or her natal family [29]. We herein aim to better understand how well the current WHO and PWDVA definitions of DV match what members of the Indian community consider constitutes DV. Study findings will help inform a culturally-tailored, country-specific operational definition of DV. A qualitative approach was chosen to provide an in-depth exploration of the perceptions of behaviors that constitute DV among both lay community members and individuals with expertise in the fields of DV and marital counseling in Pune, India.

## Methods

### Ethics Statement

This study was approved by the National AIDS Research Institute (Pune, India) Ethics Committee and the Emory University (Atlanta, USA) Institutional Review Board. Written informed consent was obtained from all subjects prior to their participation in the study.

### Study setting and context

The study was conducted in Pune, a major metropolis located in the western province of Maharashtra with a population of 3.1 million people. According to the 2011 Census of India, 62% of the population of Pune is under age 30 years, 32% live below the poverty line, and the female to male sex ratio is 0.945 [30]. The literacy rate of women is estimated at 88% and the mean age of marriage for women is 20.6 years [30].

### Study design

The study employed mixed qualitative methods: 1) key informant interviews and 2) focus group discussions (FGDs). These methods were chosen with the aim of obtaining the full breadth of the DV definition; to gain perspective of DV as defined by individuals who see the full spectrum of DV through their work with DV survivors as well as the lay community to better understand societal norms surrounding acceptance and definition of DV.

### Participant recruitment

Participants for both phases were identified and recruited with the aid of the existing National AIDS Research Institute’s (NARI) Community Involvement Plan (which involved partnering with community workers identified from the local community)[31]. Participants were contacted via telephone to establish an interview time and place convenient to them. Recruitment for the key informant interviews utilized purposive, maximum variation sampling to optimize the heterogeneity of responses related to DV experience. They were selected because of the diverse legal, social, medical, psychological, religious, and law enforcement perspectives and contexts of DV they would bring through their unique professional backgrounds. Selection criteria for the key informant interviews included being over age 18 and engagement in a profession that provides support to DV survivors. Recruitment for the FGDs utilized convenience sampling to bring both female and male lay community perspective on experience of DV. Inclusion criteria for the focus groups included being over age 18, ever-married, and female for FGD-1 and male for FGD-2. Neither key informant nor FGD participants were screened for DV or selected based on their experience of DV. Recruitment for key informant interviews and FGDs continued until data saturation was reached.

### Data collection

Both key informant interviews and FGDs were conducted by trained study staff using a standard interview guide, developed in line with the structure recommended by Hennink *et al* [32]. The interview guide was used to obtain the participant perceptions about the definition of DV, perpetrators of DV, DV examples they may have encountered through their work or acquaintances, and how DV experiences vary by culture and demographics. The guide began with the open-ended question, *“What is domestic violence among married women*?*”* Participants were further probed regarding 1) characteristics that made individuals more/less likely to experience or perpetrate DV, 2) forms and examples of DV that they had heard of (i.e. sexual, physical, emotional, verbal, control, and neglect), and 3) location and circumstance of DV occurrence. The guide was developed by ASK, pre-tested among study staff at the National AIDS Research Institute in Pune, India, and revised based on pre-testing and the feedback of the co-authors.

The key informant interviews were conducted in a private, secure room within the participant’s workplace in the participant’s language of choice (i.e. Marathi, Hindi, or English). The two gender-concordant FGDs were conducted in a community key person’s home and in a secure office at NARI, respectively, in both Marathi and Hindi. All interview and FGD sessions were followed by a debriefing session in which the audio recorders were switched off and the participant(s) were given the option to raise questions about the study, speak freely about their own or other’s experiences, and ask about DV resources. All female FGD participants were provided with the contact information for local DV resources regardless of their personal disclosure of DV as is recommended by the WHO guidelines. Key informant interviews and FGDs lasted approximate 45–60 minutes, and were audio-recorded, transcribed using Goldwave Software Version 4.19, and translated into English. Interviews were transcribed verbatim in the language in which they were recorded. Transcripts included non-verbal communication, pauses, external sounds, and fillers. Identifying information was removed from transcripts. Transcripts were reviewed by a second study member for accuracy and necessary corrections made.

### Data Analysis

The content of the transcribed, translated interviews was checked with the initial audio recording by a third individual. The transcripts were individually and repeatedly reviewed by two members of the study team (ASK and RRP). Code lists were developed both deductively from the interview guide and inductively using the principles of grounded theory [32]. These code lists were consolidated and reviewed by a third study team member (SS) for content, overlap, and clarity to generate a master code list. Investigators ASK and RPP then utilized the master code list to code the interviews and FGDs using QSR N6 Version 6.0. Discrepancies in coding were discussed between the two investigators until consensus was met. Representative quotes were selected by ASK and subsequently reviewed by all authors. Lastly the data derived from the FGDs and key informant interviews was compared with prior Indian DV literature.

### Participant Safety and Confidentiality

The safety and confidentiality protocol was established using the guidance of the WHO recommendations for conducting DV research [33]. All study field staff underwent dedicated training regarding how to ask about DV, availability of DV services, referral of participants to DV services, and optimizing staff and the participants’ safety. Interviews were conducted by female study staff, with the exception of the interview of the Islamic Mufti which was conducted by trained male study staff because of cultural considerations. All participants were offered a copy of the written consent form; however, they were encouraged to think critically before taking it home if there was concern that it would fall into the hands of a potential DV perpetrator. Key informant interviews were conducted in their respective private offices, and focus groups in private rooms in the community and National AIDS Research Institute. Interviews were halted if there was an interruption (i.e. by phone call, knock at the door). FGD participants were requested to not provide personal experiences or identifying information during the discussion, and to keep all proceedings confidential. All participants were provided with local DV and women’s empowerment organization phone numbers regardless of their responses. Consent forms with identifying information were stored in locked filing cabinets separate from similarly-secured transcripts. Electronic data was password protected. Lastly, after transcription, all interview recordings were erased from the recorders and computers, and indentifying data was removed from the transcripts.

## Results

Seventeen subjects participated in the key informant interviews (Table 1). These individuals represented the breadth of support services available for women in Pune who experience DV and frequently encounter DV survivors in their practices: a psychiatrist, gynecologist, leader of a women’s NGO, an anthropologist, human rights lawyer, the leader of a women’s association, a social worker, a sociologist, spiritual leaders representing common religions in Pune (Hindu priests, an Islamic Mufti, and a Catholic priest), an HIV counselor, a police commissioner, domestic violence lawyer, and family court counselor. Additionally, 17 lay subjects participated in the 2 gender-concordant FGDs of married individuals: 10 women (average age of 56 years, standard deviation 10 years) and 7 men (average age of 64 years, standard deviation 8 years) from a mix of joint and nuclear households and range of socioeconomic backgrounds (monthly family income of less than Rupees 2,000 to more than Rupees 10,000).

**Table 1:** Demographics of the study participants.

Interviewing Method	Occupation	Age	Gender
Key informants	Psychiatrist	55	M
	Obstetrician/gynecologist	39	M
	Women’s NGO leader	61	F
	Anthropologist	64	F
	Human rights lawyer	39	M
	Social worker	79	F
	Women’s association leader	72	F
	Sociologist	39	F
	Catholic priest	39	M
	Family court counselor	43	F
	Islamic mufti	57	M
	Hindu religious leaders	45, 42	M, M
	HIV counselor	32	M
	Police commissioner	55	F
	Domestic violence counselor	45	F
	Domestic violence lawyer	45	F
Focus group	Community women (n = 10)	ave. 56 (36–67)	F
Focus group	Community men (n = 7)	ave. 64 (50–70)	M

*ave = average*. *F = female*. *M = male*.

Several key themes regarding behaviors and acts constituting DV emerged through the key informant interviews and FGDs: 1) the exertion of control over a woman’s reproductive decision-making, mobility, socializing with family and friends, finances, and access to food and nutrition, 2) the widespread acceptance of sexual abuse and the influences of affluence on sexual DV manifestations, 3) the shaping of physical abuse experiences by readily-available tools and the presence of witnesses, and 4) psychological abuse for infertility, dowry, and girl-children.

### Exertion of power over reproductive decision-making

When asked to provide examples of acts of DV they had come across through clients or acquaintances, key informants and FGD participants frequently provided examples of the husband or mother-in-law controlling the women’s reproductive decision-making. This included control of timing and number of children a woman was to have.

A 36-year-old, married, female focus group participant: *“Now the wife is forced to have many kids*. *If there are only daughters*, *then she is forced to have another child until she bears a son*. *Somewhere the husband and the mother-in-law keep on pressuring her*. *So*, *until she bears a son*, *she keeps on conceiving even if she is going through excruciating pain*.*”*

Women’s NGO leader: *“And the woman doesn’t have the right to say that I want to undergo a family planning operation*. *I have two children*. *Enough is enough for me*. *I don’t want any more children*. *Even this decision she cannot take…A woman knows feeding more than two children or a lot more than two children is very difficult*. *She knows she cannot afford to have more children*. *Physically she cannot afford—her health doesn’t permit that*. *But even these decisions she cannot take*.*”*

And this was considered as ‘violence’ by a sociologist who stated, *“But that is violence*, *when she is not allowed to think*, *allowed to take decision regarding her bodily matters*, *giving birth to a child or not giving birth to a child—that is a form of violence*.*”*

### “Social Violence”: control over mobility, visitations, and communications

Many reported that control of a woman’s contact with her natal family and friends also constituted a form of DV. This included restricting her mobility and visits to her natal home, not permitting her family to visit her in her marital home, and stalking of communication between her and her contacts.

A 67-year-old, married, female focus group participant discussed the mobility restrictions some women encounter: *“…spending time outside is also not in the control of a woman*. *In some cases*, *their time to spend outside is limited*. *She should be back home at a specific time or she has to prepare food before he comes home*.*”*

Obstetrician/gynecologist: *“…Social violence—this is something which is I don’t think is there in the textbooks—but social violence*, *what happens*, *is that many times*, *either the husband refuses the wife to allow to meet her family*, *her parents*, *forces her to break relationships with anyone outside his own family*. *So she has to foresee*, *and she is forced to maintain the relationship only with the in-laws*. *She is supposed to black out her own parents*, *maybe her brothers*, *sisters—that is social violence*. *She is not even allowed to have a social circle*.*”*

HIV counselor describes how a patient would stalk his wife: *“‘Whom did you call*? *Who was there on phone*?*’ Like even if her parents call her*, *still he doubted her every second…He restricted her from telephonic conversations*, *would not let her to go out of the house*, *and would not allow her to go to pick up her daughter from school—this many restrictions*. *If he goes out she should not leave the house*. *He used to ask the neighbors if she goes out*, *‘When did she go*? *Where did she go*? *Did anybody come home*?*’”*

The obstetrician/gynecologist discussed how more recent technology has been used as a tool of stalking: *“And with the advent of mobile phones and tracking systems and all that stuff*, *email hacking and all these*…*these are very*, *pretty commonly used things by educated…”*

Family court counselor speaking on the changing definition of DV to encompass stalking and control of new-age communication strategies: *“Now*, *not giving a mobile [phone]*, *that ‘he won’t let me use my mobile*.*’ This is ‘violence’ too nowadays*. *Or ‘he reads my SMS [text message]*, *doesn’t give me space*, *this is violence against me*.*’ So as the state is changing*, *the definition changes as well*.*”*

### “Economic Violence”

Several key informants also reported their clients experiencing financial control. This included having power over the woman’s earnings, restricting her access to family income, and her access to *stridhan*.

A women’s NGO leader speaks of the personal experiences of the social workers within her organization: *“Whatever he [the husband of a social worker] earns temporarily*, *temporary worker*, *or whatever he*, *periodically*, *sporadically*, *whatever he earns*, *he has full right over that income*. *That doesn’t form a part of the family income*. *He has…he does what he likes with that*. *Whereas*, *whatever the woman earns becomes the family income*, *including his own needs to drink*, *to booze…so she supports that*.*”*

Family court counselor: *“If we look into the financial type [of domestic violence]*, *then even if the wife works [outside the home]*, *all of her salary goes to the husband*. *And therefore*, *she has no economic independence*. *If sometimes she feels like giving some money to her maher [her own parents]*, *[like] if her mother is sick*, *father is sick*, *then how can [she] spend her own money*? *She cannot spend her money on her own*, *so due to that*, *financial harassment occurs*. *And if she is not earning*, *then she won’t have any money at all*. *That becomes a form of financial harassment*.*”*

DV lawyer: *“Many a times*, *there is stridhan [the gold or silver ornaments that are given by the girl’s parents at the time of wedding]*, *stridhan that she brings*, *many ornaments at the time of wedding*, *but all those are taken away and kept in the cupboard*. *When the mother-in-law says*, *only that day those should be worn*. *So that all falls under ‘economical violence*.*’ Or they dispose of her wealth and her salary on their own…*.*”*

### Access to food and nutrition

Some participants discussed that marital family members also exerted power over a woman’s dietary preferences or her access to fresh food and that these behaviors constituted DV.

Family court counselor: *“Like if at her parents’ [home] they do not eat non-veg [meat] at all*, *and if she has to make it after she goes to her husband’s place*. *And if she is forced to eat it*, *although she still doesn’t want to*, *then that too is emotional harassment*. *She has to do it against her will*. *She has to do it against her beliefs*. *That is what we call ‘violence*,*’—that which is happening against her will*.*”*

An Islamic Mufti spoke of a case where the husband prioritized his own family’s access to food over his wife’s: *“[The husband would tell the wife]*,*‘see my father*, *etcetera*, *will eat good food but you have to eat this stale food*. *My mother*, *my father*, *my brothers and sisters will not eat the stale food*, *you have to eat it*.*’”*

### Sex versus sexual abuse: acceptance, under-recognition, and influence of affluence

Case reports of sexual abuse were reported by several key informants, many of whom discussed the widespread acceptance of sexual DV.

Women’s NGO leader: *“[If] the woman doesn’t want to have sex*, *and she is forced into it by the husband*, *it is domestic violence*. *But unfortunately*, *in India*, *women do not look at it like this*. *This is violation of their rights to be assertive about sex*, *they don’t even look at it like violation*. *You talk to ten women and nine will say that that is his right to have sex*, *to demand sex*, *and when he wants*, *whether the woman wants it or not*, *is his right*.*”*

Women’s NGO leader: *“She’s living in her mother’s house*. *Her brother is married*. *His children*, *her two children*, *and her husband—everyone lives in that house*. *She says that in the evening*, *whenever he wants*, *he just takes me*, *drags me*, *takes me inside [the] room and closes the door*. *In front of everyone*, *sister*, *I feel like I’m dying*. *Dying at 5 o’clock*, *6 o’clock*, *7 o’clock*, *5*:*30…whenever he wants it*. *He just closes the door from the inside*. *He makes me feel like I’ve died and died*.*”*

Similarly, a family court counselor explained her interaction with an abusive couple she counseled through her work: *“…so she always used to say that he would not even leave me during the four days of my period*. *Regularly*, *every day he wants sex*. *So I was trying to make him [the husband] understand during counseling…So*, *in that*, *he said to me that a ‘wife is just like toplayatli bhakar [bread in a basket]*.*’ Whenever he wants*, *he can just break it and eat it*. *Whenever I want my wife*, *I should be able to have her—and that’s the meaning of a marriage*.*”*

Human rights lawyer: *“Then sometimes in such relationships we have seen that the father-in-law eye his daughter-in-law [with sexual intentions] and he wants to have illicit relations with his own daughter-in-law*. *People are like that too*. *Then everyone together they hit her*. *And whether educated or non-educated*, *it is happening in all sectors*.*”*

Some participants discussed how sexual abuse was particularly common among couples employed in the information technology sector, one of the largest industries in Pune. They attributed this to a large disposable income allowing for the purchase of pornography (or blue films).

Human rights lawyer: *“But in information technology*, *computer sector*, *I think it is my personal opinion*, *that they are earning out of proportion and because of that what happens is they do not have money as an issue or problem*. *So as money is not an issue they keep on experimenting things*. *I mean*, *there is a relationship with it*, *experimentation and money*. *So then they watch different blue films [pornography]*, *different sex videos*, *sex films*, *and then they expect their wives to perform like that…there was a man*, *he made his own rule in the house*. *Only two of them used to stay there*. *He made the rule that all the windows should be closed and she should do everything in a naked situation*. *Everything*. *She should not wear clothes*. *And if she would wear clothes*, *then he would hit her*, *that ‘did you ask me*? *Why did you wear clothes*?*’ and whenever he felt like doing sex*, *he can do anywhere*. *So sometimes in the kitchen*, *the other time somewhere else*, *anywhere*.*”*

Family court counselor: *“Then in many cases*, *blue films [pornographic films] are shown*, *and [the women] are asked to replicate that*. *And in some high-profile [settings] group sex—they are forced to perform this*. *And there are some cases that have come to us where the husband takes [his wife] and asks her to have sex with another person in front of him*. *Even if that woman does not want that*, *she is forced to do it*. *In some cases*, *I have come across such examples in which the husband had a fantasy to video shoot the actual act during sexual intercourse*, *and he used to video shoot it in the mobile [cell phone]*, *in the mobile [camera] he used to shoot it*.*”*

### Experiences of physical abuse: shaped by readily available tools and the presence of others

Physical abuse was also reported, although none of the participants shared stories of abuse in which guns or knives were used as weapons of abuse. Rather, severe forms of abuse included burning and use of more readily available tools such as sticks.

A 63-year-old, married, female focus group participant: *“They are arm-twisted because of dowry*. *For example*, *if they have received less dowry*, *then they torture her to get more money*. *She is ill-treated*, *beaten*, *or even in Maharashtra*, *many cases have occurred when she was burnt alive or poisoned*, *and her parents aren’t even made aware*. *Then they inform them that your daughter has suddenly died*. *If they get the money*, *then there won’t be any problems*, *but they would ask for money again*.*”*

Human rights lawyer: *“The woman was bedridden because of the burn injuries*. *She was attempted to be murdered by burning her*. *And see the whole disfigured body that her skin had stuck to even the hand*, *even her hand was unable to be taken out*, *it was stuck to the body as all her skin had melted…She took her thumb impression on the authority letter [since] she was not in a position to sign*. *Her thumb was burnt*. *It was taken like that*, *all blood and ink together*. *I mean*, *even judge asked us that*, *‘how did you take like this red-colored thumb impression*, *isn’t that lady educated*?*’”*

The perceived severity of abuse experienced seemed to be influenced by whether others were present to witness the event. For example, a women’s NGO leader gave particular importance to a woman being slapped by her husband when others were there to observe them: *“‘He slapped me*, *he hit me in the head*, *in front of 4 people he used abusive language*,*’—he pulled her sari in the bazaar and yelled at her*. *This is all abuse—isn’t it*?*”*

Physical eviction of a woman from her own marital home was also noted by several participants.

An HIV counselor described: *“Even such cases are there where they were beaten up*, *kicked or beaten with the sticks*, *or slapped in the face*, *or thrown out of the house at odd hours—even such cases are there*.*”*

Family court lawyer: *“they are thrown out of the house*. *For any damn reason*, *you get out*, *you didn’t bring this*, *that due to such reason they throw her out of house*, *or if her behavior is not liked or if she doesn’t know how to cook*, *there was more salt in the food*, *due to many such reasons the women are thrown out of the house*.*”*

### Psychological abuse: humiliation and mental torture for infertility, dowry, and girl-children

Many also discussed how women commonly experience psychological abuse or are shunned by their marital families, whether for lack of provision of sufficient dowry or wedding-related gifts, infertility, or delivery of a girl-child.

Family court counselor: *“There are many arguments over the give and take [dowry]*. *Now we are heading towards 21* ^*st*^ *century*, *but still in many cases we are seeing…like dowry and all doesn’t exist*, *but still*, *your parents did not give this in the marriage’ [or] ‘the wedding should have been done like this’ [or] ‘they did not do our maan-paan [/providing the groom’s family members special respect and gifts during the wedding/]*.*”*

A 69-year-old, married, male focus group participant further elaborated: *“She thinks*, *‘if I open my mouth then these people [her in-laws] would torture me more*.*’ So she keeps quiet and goes to her parents and tries to get the money*. *If she doesn’t get the money*, *then she would be tortured more*, *even beaten*.*”*

The family court counselor explained a case she encountered of a woman being verbally tormented by her mother-in-law for being infertile: *“There was one such case that had come to me in which the woman could not have a child*. *They had tried a lot*, *but they could not conceive a child*. *And the mother-in-law used to blame her for everything*. *There was a vine in their garden*, *and that vine never produced flowers*, *so she was always used to say*, *‘throw away that infertile vine*. *It should not be kept in our garden*. *Throw away that infertile vine…’ Now that is a kind of indirect emotional harassment*. *And she came to realize that ‘this is being said to me*.*’ That ‘throw her out of the house*, *throw her out of the house*,*’ this is what her mother-in-law wanted to tell her son*, *but each time she would give the example of the vine*. *And because this [taunting] became more frequent*, *she too began feeling that ‘we are not able to bear a child*, *so it’s my fault*. *Mother-in-law is also harassing us*.*’ …When the sister-in-law delivered a baby and she went to the naming ceremony*, *she was not allowed to touch the baby because she [was] infertile*.*”*

67-year-old, married, female focus group participant: *“There are some who torture their wives because she has only daughters*. *They never consider that even they [the husbands] are equally responsible*. *My own sister’s example*, *still as in*, *even after her daughters are educated*, *they are now engineers*, *and they are married to good husbands—still my sister is berated*.*”*

Obstetrician/gynecologist: *“I have seen a woman delivering a girl-children and the husband*, *the in-laws*, *never visiting them*. *If they visit*, *they are too harsh on the mother*, *they criticize her*, *they make her repent*, *make her cry*, *make her feel that she has done a big crime*.*”*

## Discussion

This qualitative study provides a detailed, comprehensive understanding of community perceptions regarding the definition of DV in the Indian context. The key informant interviews explored expert knowledge and beliefs regarding behaviors that constitute DV and allowed for in-depth exploration of individual case stories. The key informants, shaped by the diversity of cases they encounter through their varied professions, shed light on different legal, religious, cultural, psychosocial, and medical aspects of the definition. In contrast, the focus groups offered insight into broader, lay community views through provoking discussions regarding behaviors constituting DV and explanations of conflicting perspectives. A comprehensive, culturally-tailored definition of DV emerged that encompassed forms of physical, sexual, and psychological abuse as well as control. Exertion of control over a woman’s reproductive rights, financial expenditure, her socialization with friends and family, and access to food and nutrition were raised by many participants as being dimensions of the Indian definition of DV.

While many central elements of DV are universal, our study findings suggest that certain manifestations of abusive behavior in India differ because of its culture, evolution of technology, and material availability (Table 2). For example, the traditional Indian marital family structure may protect against, but also fuel some manifestations of DV such as abuse perpetration by in-laws, threats or acts of evicting a woman from her marital home to return her to her natal family post-marriage, and abusing the woman for insufficient material assets provided by her natal family at the time of marriage to herself and her new marital family to cover her expenses. Deeply-rooted values for having children after marriage and preference for male children result in women suffering abuse for experiencing infertility and delivering girl-children. Misinterpretation and misuse of religion were also cited in perpetuating abuse; for example, through restricting a woman’s access to food and forcing her to change her dietary preferences. Interestingly, while gun violence was never reported as a manifestation of severe physical abuse, burning, poisoning, and stick-beating were reported with frequency, probably reflecting the relative ease of availability of such tools. Lastly, many study participants implicated India’s technology revolution for yielding novel forms of DV through forcing women to replicate humiliating sexual acts performed in increasingly-viewed pornography, as well as stalking partners through text messages and social networking sites.

**Table 2:** Comparison of the definitions of DV provided by the World Health Organization, the 2005 Protection of Women against Domestic Violence Act, and study participants enrolled in Pune, India.

	2013 WHO systematic review	2005 WHO MCSWHDV	PWDVA definition	Study findings
**DV perpetrator(s)**	Intimate partner	Intimate partner	Member of a shared household	Any member of the marital family
**Time period**	Since age 15	Lifetime, 12 months	Lifetime	Since marriage
**Forms of DV surveyed**				
Physical	Yes	Yes	Yes	Yes
Sexual	Yes	Yes	Yes	Yes
Psychological	No	Yes	Yes	Yes
Economic control	No	No	Yes	Yes
Non-economic control	No	Yes	No	Yes
**Acts of physical abuse**	kicked, dragged or beat her	kicked, dragged or beat her	Not specified, defined in terms of inflicted harm	kicked, dragged or beat her
	choked or burnt her	choked or burnt her		choked, burnt, or poisoned her
	slapped her, or threw something at her that could hurt her	slapped her, or threw something at her that could hurt her		slapped her, or threw something at her that could hurt her
	pushed or shoved her	pushed or shoved her		pushed or shoved her
	hit her with a fist or something else that could hurt	hit her with a fist or something else that could hurt		hit her with a fist or something else that could hurt
	threatened her with, or used a gun, knife or other weapon against her	threatened her with, or used a gun, knife or other weapon against her		threatened her with or used a sharp weapon: broken glass, razor blade, knife
				threatened her with or used a blunt weapon: belt, broomstick, stone, rolling pin
				made her work excessively
				twisted/pulled her hair
**Acts of sexual abuse**	physically forced to have sexual intercourse against her will	physically forced to have sexual intercourse against her will	Not specified, defined in terms of inflicted harm	physically forced to have sexual intercourse against her will
	having sexual intercourse because she was afraid of what her partner might do	having sexual intercourse because she was afraid of what her partner might do		having sexual intercourse because she was afraid her partner would harm someone she cared about
	forced to do something sexual that she found degrading or humiliating	forced to do something sexual that she found degrading or humiliating		forced to do something sexual that she found degrading or humiliating (i.e. replicating pornographic film, videotaping intercourse)
				physically forced to have sex when she was menstruating
				forcing her to have sex with someone else
				made her drunk or high to force her to have sex
				forcing her to have sex without a condom
				intentionally performed sex forcefully to harm her
**Acts of psychological abuse**	Not included	being insulted /made to feel bad about oneself	Not specified, defined in terms of inflicted harm	being insulted /made to feel bad about oneself
		being humiliated or belittled in front of others		being humiliated or belittled in front of others when alone
		being threatened with harm (directly or indirectly by threatening to hurt someone she cared about)		being threatened with harm (directly or indirectly by threatening to hurt child or natal family member)
		being intimidated or scared on purpose		eviction from marital home
				harassment for dowry or *maanpaan*
				belittling about her poor health
				threatening to remarry
				spreading false rumors
				starving her or providing her stale food
				taunting her for a girl-child, infertility
				forcing dietary change (vegetarian->non-veg)
				forcing her to fast
**Acts of control**	Not included	Social restrictions (contact and visitations with family, natal family, knowledge of her location)	Economic or financial deprivation or control (including dowry, *stridhan*, property, expenditure)	Economic or financial deprivation or control (i.e. dowry, *stridhan*, property, expenditure)
		Angered if she speaks with other men		Social restrictions (contact and visitations with family, natal family, knowledge of her location)
		Accusing of infidelity		Angered if she speaks with other men
		Ignore/treat indifferently		Accusing of infidelity
		Control health care access		Ignore/treat indifferently
				Control health care access/medications
				Control of her reproductive rights and contraception
				Control of her employment choice
				Control of her choice to relax/rest
				Confining to the home; not allowing her to attend social events
				Control of her appearance
				Ignoring her sexual desires

Thus, while the WHO definition serves as an essential starting point to develop an operational DV definition for India, our results stress the need for it to be culturally-adapted. For example, although the WHO acknowledges DV can be perpetrated by any member of the household, current WHO studies limit the definition to abuse perpetrated by intimate partners—not other marital family members. While the purpose of doing so is likely to make meaningful cross-country comparisons, it runs the risk of dangerously underestimating DV experienced by women in India. Second, although not explored in our study, the 2013 WHO systematic review definition surveys abuse occurring after age 15, but may fall short in capturing early experiences of DV for the 27% of Indian women who are married before then [34]. Next, it was limited to physical and sexual abuse because of the ‘lack of consensus’ on control and psychological violence measures; however, psychological abuse and control were mentioned by several study participants as an important, frequent, and devastating aspect of DV experienced by Indian women. Shortcomings of the WHO definition in evaluating psychological abuse and control in the Indian context include: 1) economic control (i.e. over *stridhan*, property, and the couple’s earnings) and psychological abuse over dowry or *maanpaan (gifts and special respect provided to the groom’s family members during a wedding)*, 2) “new-age” social control, stalking, or psychological abuse via text messages and social media, 3) control over reproductive and contraceptive rights and psychological abuse for infertility or delivering a girl-child, 4) confinement to, or alternatively, eviction from the marital home, and 5) nutritional deprivation or control (i.e. through enforcing fasts and forcing dietary change away from vegetarianism against religious beliefs). The WHO definitions are comprehensive in regard to physical and sexual DV; however, specific mention of the commonly-used tools to inflict physical abuse (that women may not perceive as weapons) may improve the sensitivity of the physical DV definition. Similarly, surveying forced sex during times perceived as sexually-humiliating like menses in addition to surveying humiliating sexual acts may enhance sensitivity of the sexual DV definition. We thus propose to expand the Indian operational definition of DV to include physical, sexual, and psychological abuse, stalking, and economic and other forms of control perpetrated against a married woman by her spouse or other members of the family in which she marries. Surveys that stem from this definition should consider inclusion of the various aforementioned forms of DV (summarized in Table 2).

Our findings also have several implications for DV prevention interventions and policy development in India. First, prevention strategies should incorporate increasing awareness of the many forms of abuse that constitute DV beyond physical abuse alone and emphasize that the PWDVA offers protection against many of these. Second, they should consider incorporating content that heightens knowledge that infertility and fetal sex determination is a biologically-driven phenomenon out of the woman’s control. Third, they should teach couples the difference between sexual intercourse and marital rape, and engage them to discuss means of ensuring that sexual intercourse is enjoyable for both. Additionally, perpetrator intervention programs should consider engaging not only husbands but other members of in the in-law family.

Our study possesses some limitations. First, we limited our participant pool to Pune, an urban, well-developed metropolis, in the western state of Maharashtra. In doing so, we may have not captured the DV experiences of those in rural and tribal communities, or other regions of India where DV may manifest differently[35]. Similar themes have, however, been discussed in prior DV literature in India from other urban and rural parts of India. Second, some may view limiting our sample to two FGDs with community members as a shortcoming; however, upon triangulating the emerging themes from focus group data with that of the key informant interviews, the three researchers involved in the analysis (ASK, RRP, and SS) felt data saturation had been reached. Additionally, we do not capture the DV experiences of young, newly-wed women since the average age of participants in both FGDs was over 50 years. We chose not to purposively include younger subjects in the FGDs—and rather allowed for participants from a homogenous age group—to foster comfortable group dynamics in the discussion. We did not conduct additional FGDs of younger, newly-wed subjects because we felt the key informants, who often work with young, newly-married women, brought this perspective. Also, we did not sufficiently explore DV among women in cohabiting relationships because of the low frequency with which these relationships occur in the Indian context. Thus, we cannot comment on whether the Indian DV definition, like the WHO definition, should include this population. Lastly, although we are confident that some of the issues raised in the study are specific to India, it is possible that the expanded DV definition put forth by our findings may be more applicable than current WHO operational definitions to other low- and middle-income countries with high DV prevalence. For example, recently emerging literature from other countries within South Asia suggest that themes like perpetration of DV by in-laws, emotional abuse for infertility, dowry, or failure to have a male son, the use of sticks and kerosene-burning to perpetrate physical abuse, deprivation of a woman’s access to the material assets given to her at the time of marriage by her parents, and forcing sexual intercourse with others are not unique to India [36–40]. Future research should examine the extent to which local definitions of DV vary from the definitions provided by the WHO.

In conclusion, our study emphasizes the need to broaden the surveillance definition of DV in India to truly capture the abuse and control experienced by women at the hands of their marital family. The findings not only add to the literature informing the development of future culturally-tailored DV interventions, but also call for an India-specific measure to better quantify DV and evaluate the efficacy of such interventions.
